# An innovative methodology for the non-destructive diagnosis of architectural elements of ancient historical buildings

**DOI:** 10.1038/s41598-018-22601-5

**Published:** 2018-03-12

**Authors:** Silvana Fais, Giuseppe Casula, Francesco Cuccuru, Paola Ligas, Maria Giovanna Bianchi

**Affiliations:** 1 University of Cagliari, Department of Civil and Environmental Engineering and Architecture (DICAAR), Cagliari, 09123 Italy; 2 Istituto Nazionale di Geofisica e Vulcanologia, Sezione di Bologna, Via Donato Creti 12, Bologna, 40128 Italy

## Abstract

In the following we present a new non-invasive methodology aimed at the diagnosis of stone building materials used in historical buildings and architectural elements. This methodology consists of the integrated sequential application of *in situ* proximal sensing methodologies such as the 3D Terrestrial Laser Scanner for the 3D modelling of investigated objects together with laboratory and *in situ* non-invasive multi-techniques acoustic data, preceded by an accurate petrographical study of the investigated stone materials by optical and scanning electron microscopy. The increasing necessity to integrate different types of techniques in the safeguard of the Cultural Heritage is the result of the following two interdependent factors: 1) The diagnostic process on the building stone materials of monuments is increasingly focused on difficult targets in critical situations. In these cases, the diagnosis using only one type of non-invasive technique may not be sufficient to investigate the conservation status of the stone materials of the superficial and inner parts of the studied structures 2) Recent technological and scientific developments in the field of non-invasive diagnostic techniques for different types of materials favors and supports the acquisition, processing and interpretation of huge multidisciplinary datasets.

## Introduction

The diagnostics of cultural heritage is a very time consuming and delicate task. The need for the conservation of the monumental structures of the Cultural Heritage has led to a significant development of multidisciplinary non-destructive diagnostic investigations. With non-destructive tests it is now possible to obtain all the qualitative and quantitative parameters needed to plan the recovery and preservation of a monumental structure.

The reliable interpretation of the multidisciplinary non-destructive diagnostic techniques requires a good knowledge of the petrographical characteristics of the building stone materials. Therefore, one research aspect that needs to be improved is the efficient integration of the petrographical information into a model of the investigated structure deduced from the multidisciplinary non-destructive techniques. Nowadays optical and electron microscopy are very useful techniques for the knowledge of the intrinsic characteristics of the stones used in the construction of monumental compounds and their potential degradation.

The compositional and textural characteristics of a rock play a fundamental role in the interaction with the external environment. For example, in carbonate rocks such as those studied in this paper, the knowledge of the rock texture is a very important aspect^1–3^ because it depends on significant elements such as the grain disposition and the presence of matrix or cement as binding materials. These features, typical of each lithotype, affect the amount of pores and their geometry (size, shape and disposition). In fact, the porosity is strictly related with the granulometric features, such as the grain packing arrangements. Scanning Electron Microscopy (SEM) analyses especially can give an evaluation of the microscopic characteristics of the rocks, such as type of micropores and their connection within the matrix, which favor water retention and, thus, their chemical weathering and physical disintegration. The development of an integrated methodology for the systematic diagnosis of the conservation status of monuments could lead to the detection of the material properties on the shallow and inner parts of the monumental structures under investigation.

In the workflow here presented, starting from a depth knowledge of the petrographic characteristics of the carbonate stone materials, a survey with 3D Terrestrial Laser Scanner (TLS) methodology is combined with ultrasonic measurements in the characterization of the building stone materials following a multi-step procedure. In the diagnostics of monuments, proximal sense techniques aimed at the 3D modeling of the complex shapes of ancient artifacts can be implemented and performed together with surface geometric anomalies and reflectivity patterns of the building parts of the studied elements by means of modern TLS capable of positioning and monitoring millions of point clouds in a few minutes. With the aid of object-oriented modern software a 3D model of monuments or architectural elements such as walls, columns, pillars, facades etc. is possible using the aggregated registered unstructured point clouds deriving from the structured ones acquired in a TLS survey. Millimeter or sometimes sub-millimeter accuracies can be reached in the position of the points of the prospects of the targeted objects in order to appreciate geometrical anomalies typically present in the architectural elements of ancient buildings.

In this work the 3D TLS technique was integrated with the *in situ* ultrasonic measurements for the characterization of the building stone materials. Nowadays, the non-destructive ultrasonic method represents one of the most reliable methods used in the diagnostics of the stone building materials of monumental structures. The close relationship between the propagation velocity of the ultrasonic pulses in the stone materials and their physical, textural and mineralogical features is the strength of this method. Thanks to the integrated analysis of the ultrasonic method with the physical and petrographical data of the stone building materials and with parameters from various “non-destructive” techniques such as Infrared Thermography (IRT)^4–6^ and TLS^7–11^, it is possible to obtain important information about the conservation status of the monumental structures. Furthermore, from the observation of the changes in time in the elastic properties of the stone materials by ultrasonic measures, it is possible to identify the evolution of the degradation of building materials and provide useful information about the effectiveness of the restoration works. As a matter of fact, alterations in the stone materials normally cause a decrease in the ultrasonic longitudinal pulse velocity values, which can be considered representative of their elasto-mechanical behaviour.

Moreover, ultrasonic pulse velocity testing carried out with different data acquisition and processing techniques has been done by several authors^1,8,12–15^ as a useful and reliable non-destructive test in assessing the elasto-mechanical characteristics of stone materials, such as the dynamic modulus of elasticity (Young’s modulus) and the uniaxial compressional strength, and in reconstructing the internal characteristics of architectural elements. In particular, with the three-dimensional acoustic data obtained with the ultrasonic tomography technique, it is possible to visualize the internal conditions of the building stone materials of the investigated architectural elements in 3-D space. Correlations between the petrographical characteristics, in particular texture and porosity, of the materials and the propagation of the ultrasonic longitudinal signals (P - waves) are also analyzed in order to improve the interpretation process^2,11^. In fact, knowledge of the textural characteristics such as pore geometry can facilitate the understanding of the longitudinal ultrasonic signal behavior through the stone materials.

The use of the above multi-step procedure can have a considerable impact in defining the conservation status of the investigated structures in their shallow and inner parts making stone conservation treatments more cost-effective. Moreover, this methodology can be used to re-treat the crack pattern of buildings affected by historical seismicity in regions characterized by a high seismic risk.

In synthesis our workflow starts with the analysis of the thin sections of the studied stone materials by means of optical and electron microscopy. This is very important to reconstruct the pore systems that in carbonate stone materials like those analyzed in this study usually contain both pores and inter-connections between pores.

Once the first step is completed we follow up with the second one which consists of the 3D modeling of the artifact or parts of them by TLS.

**Figure 1:**
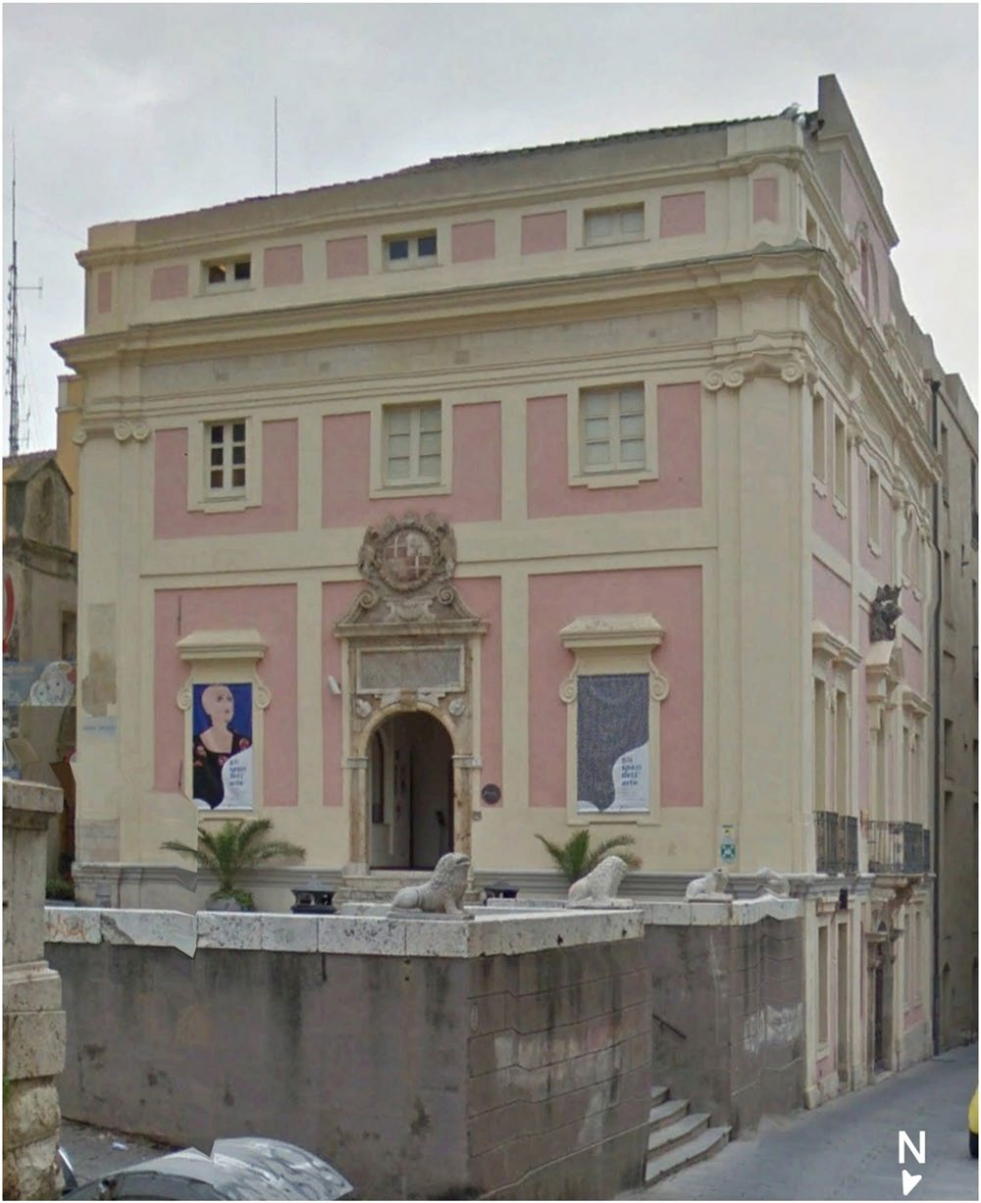
Aerial view of the *Palazzo di Città* building in the historical centre of Cagliari, which was town hall from the Middle Ages to the early twentieth century.

The problem of the TLS technique is that despite its high productivity it can only give information on the surfaces of investigated objects, while the acoustic diagnostic methods depending on the frequency used for the emitted pulse and on the data acquisition technique, can give information on their deep internal parts. Therefore, in the final step of our procedure, starting from the 3D terrestrial laser scanner results, the *in situ* acoustic survey in the 24 kHz–54 kHz ultrasonic range was planned and carried out by collecting and processing the ultrasonic data (namely P-wave travel times) with different data acquisition and processing techniques. The ultrasonic parameters, especially longitudinal velocity (Vp), can be measured very accurately starting from the *in situ* observations and correlated with various material properties with reasonable confidence. Use of the ultrasonic longitudinal signal (P-wave) in the velocity detection instead of the transverse signal (S-wave) is recommended in the *in situ* inspection, since the first arrival of the ultrasonic signal can be interpreted unambiguously as a compressional wave along the shortest accessible acoustic path. The task of this third step in the integrated procedure is to compare both the petrographical rock properties with the elastic ones, and the TLS geometrical anomalies with the anomalies of the velocity field detected with the ultrasonic methodology. What is very important in the described procedure is the integrated multi-stepped application and the subsequent accurate comparison of the results of the three above mentioned techniques. The integrated interpretation of the independent data from different sources with a different physical meaning helps to overcome ambiguities in the interpretation of individual datasets and contributes in increasing the level of confidence of the integrated interpretation. This paper describes the application of the proposed multi-step procedure aimed at verifying the conservation status of an architectural element of historical relevance of the *Palazzo di Città* historical museum (Fig. 1) in the old town of Cagliari (Italy).

## Results and Discussion

In this section we underline the results of the integrated application of three diagnostic techniques like petrography complemented by SEM and OM (Optical Microscopy) analyses, TLS and ultrasonic analyses of the materials used in assembling artefacts. The analysis of the thin sections of the study carbonate rocks (*Pietra Forte*, *Tramezzario* and *Pietra Cantone*) using optical and electron microscopes has provided important information about their compositional and textural characteristics, as well as the porous system on which their susceptibility to degradation depends.

The observations of the thin sections reveal that *Pietra Forte* can be defined as boundstone^16^ or Lithothamnium biolithite^17^, prevalently made up of rounded – sub rounded algal rhodoliths.

**Figure 2:**
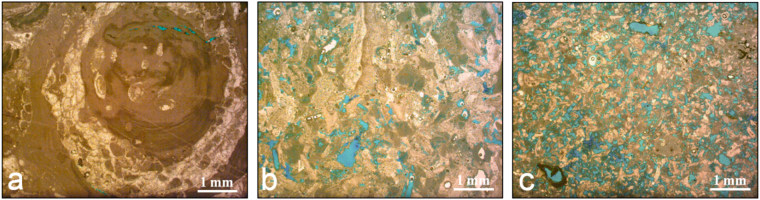
Thin section of the three carbonate lithotypes, stained with the epoblue dye: (**a**) *Pietra Forte*, (**b**) *Tramezzario*, (**c**) *Pietra Cantone*. Images under polarizer only.

*Tramezzario* can be defined as grainstone^16^ - packstone^16^ or biosparite^17^ with bioclasts, prevalently made up of well sorted, sub rounded - subangular algal rhodolith fragments and mollusk macrofauna; their sizes ranging between 0.05 mm–4.00 mm. *Pietra Cantone* may be defined as mudstone^16^ – wakestone^16^ or biomicrite^17^, with well sorted sparse bioclasts (sizes ranging between 0.10 mm e 2.5 mm), prevalently made up of foraminifera. The different relationships among grains-matrix or grains-cement, bioclasts packing and their degree of rounding highlight various typologies of porosity. For a better identification of the pore network, the thin sections of the above carbonate materials were stained with an epoblue resin^18^ (Fig. 2).

The porosity detected at the optical microscope can be classified as mesoporosity^19,20^. In *Pietra Forte* (Fig. 2a), mesoporosity is about 1–2%, prevalently secondary (intraparticle and fracture types), at times it may be of the primary type (algal growth framework porosity). In the *Tramezzario* (Fig. 2b), mesoporosity is secondary, about 5–15% and prevalently of the channel, intraparticle, moldic and fracture types. The mesoporosity of *Pietra Cantone* (Fig. 2c) is mainly of the primary type (interparticle types); its percentage is 7–25%.

**Figure 3:**
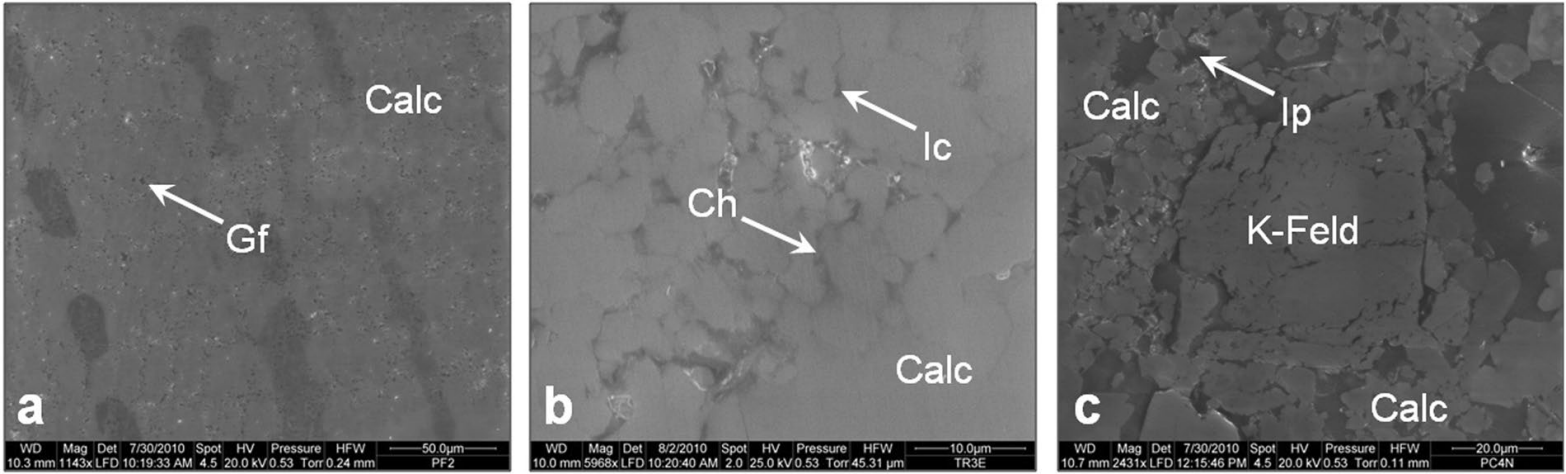
SEM analysis of the three carbonate lithotypes: (**a**) *Pietra Forte*, (**b**) *Tramezzario*, (**c**) *Pietra Cantone*. Calc (calcite); Gf (Growth framework porosity); Ic (Intercrystal porosity); Ch (Channel porosity); Ip (Interparticle porosity); K-Feld (Potassic Feldspar).

From the SEM analysis it was pointed out that *Pietra Forte* is exclusively made up of calcite, characterized by single subhedral microcrystals (2–3 μm) or polyhedral aggregates of calcite. An important micropore network connected mainly with the laminae of algal growth is present. At the microscale this network is characterized by growth framework (Fig. 3a) and intercrystal types^19^, of pore dimensions smaller than 5 μm.

The *Tramezzario* is made up of single subhedral - anhedral microcrystals (2–3 μm) or polyhedral aggregates of calcite. Two categories of micropores are present: i.e. the interparticle type (micropore sizes ranging from 5 μm to 10 μm) and the intercrystal type^19^ with sizes between 4 μm and 5 μm (Fig. 3b). Often the pores are filled with micrite crystals not relating to a depositional environment, but reflecting the disintegration of the bioclasts.

The *Pietra Cantone* shows a more complex pore network and composition. Besides the carbonatic component made up of foraminifera and micrite grains, with sizes ranging between 1 μm from 4 μm, a small amount of terrigenous minerals, such as quartz, feldspar and micas is also present. Microporosity, related to the carbonate mud, is interparticle (mean pore size about 2 μm) (Fig. 3c).

The effects of the above described textural characteristics of the rocks on the ultrasonic longitudinal velocity propagation, especially the grain size, the different types of porosity and the presence of cement or matrix among grains or pores were established and it was found that the relationship between these properties changes as the carbonate rocks become weathered. Starting from the knowledge of the petrographical characteristics of the carbonate building materials of the investigated architectural structure it is possible to better analyze the results of the non-invasive techniques applied here (TLS and ultrasonic techniques). In fact, any supplementary information of a different nature can contribute to improve the reliability of the diagnostic process. The robustness of the diagnostic process can increase if it is based on heterogeneous experimental data.

**Figure 4:**
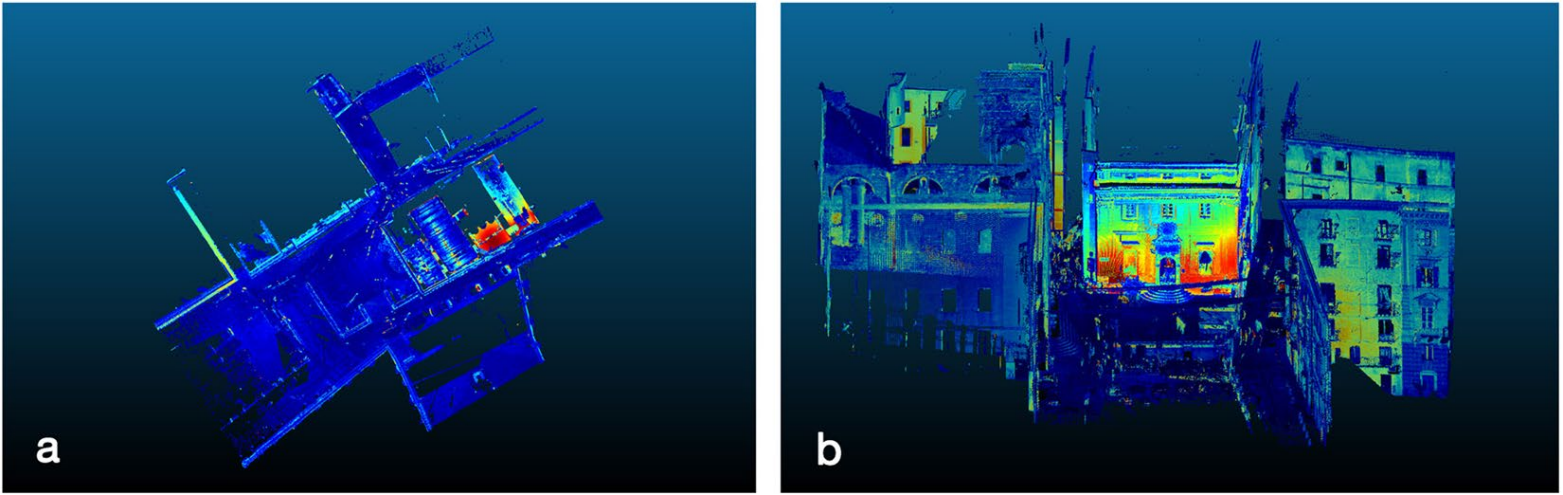
Aggregated reflectivity map of the *Palazzo di Città* monumental compound in the city centre of Cagliari. (**a**) ortographic and (**b**) perspective view. TLS data elaboration with JRD 3D Reconstructor® (http://www.gexcel.it/it/software/jrc-3d-reconstructor), and CloudCompare (http://cloudcompare.org).

Regarding the TLS results, as previously described, the main goal of the pre-processing and processing steps of data acquired with TLS methodology is a noise-filtered, unified unstructured point cloud of a complex shape representing the 3D model of the ancient building and museum of the *Palazzo di Città* (Fig. 4).

**Figure 5:**
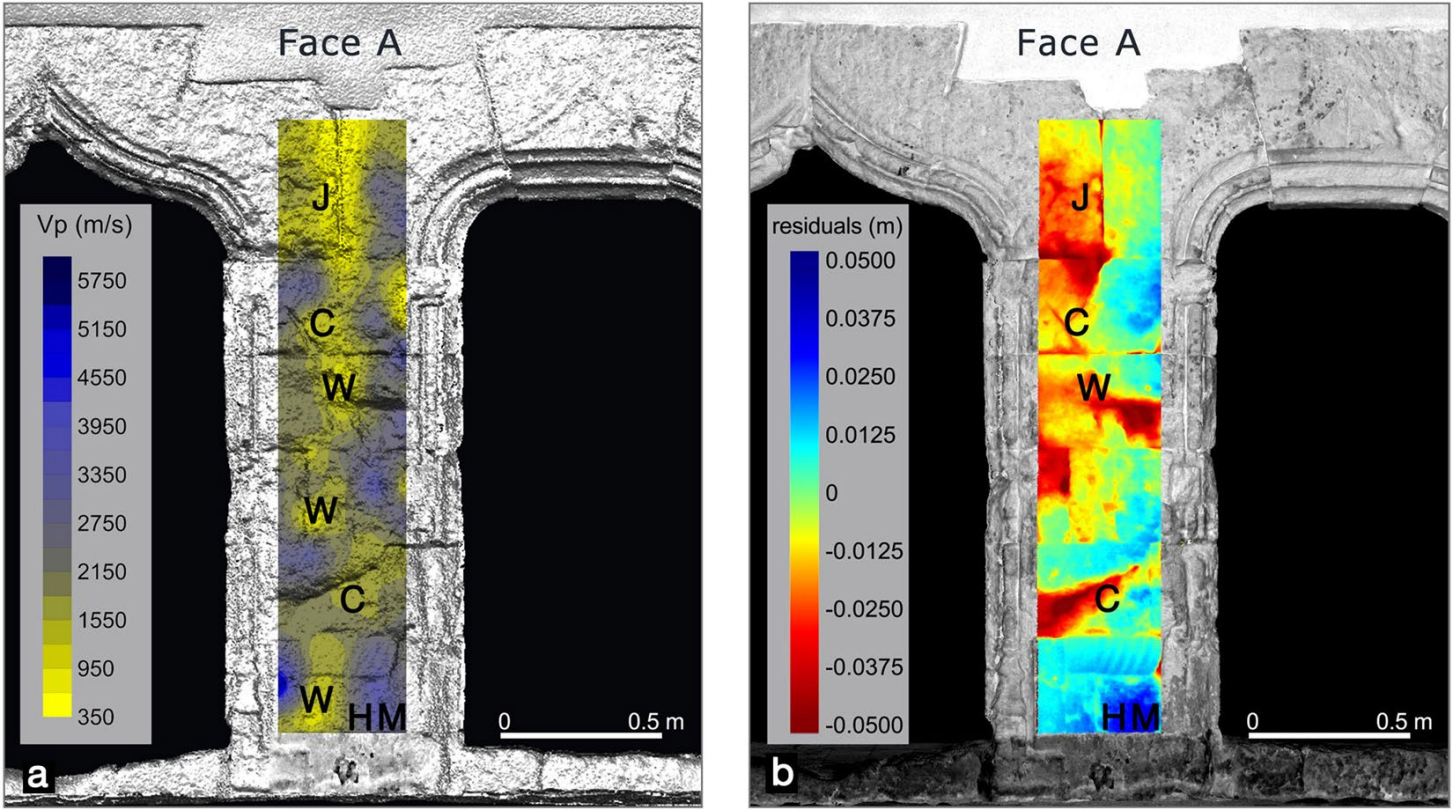
Comparison between the ultrasonic map and the TLS results on face A of the pillar. The two techniques of the NDT survey show matching results highlihted by uppercase letters (C = crack; J = Junction; W = Weathering; HM = Health Material).

**Figure 6:**
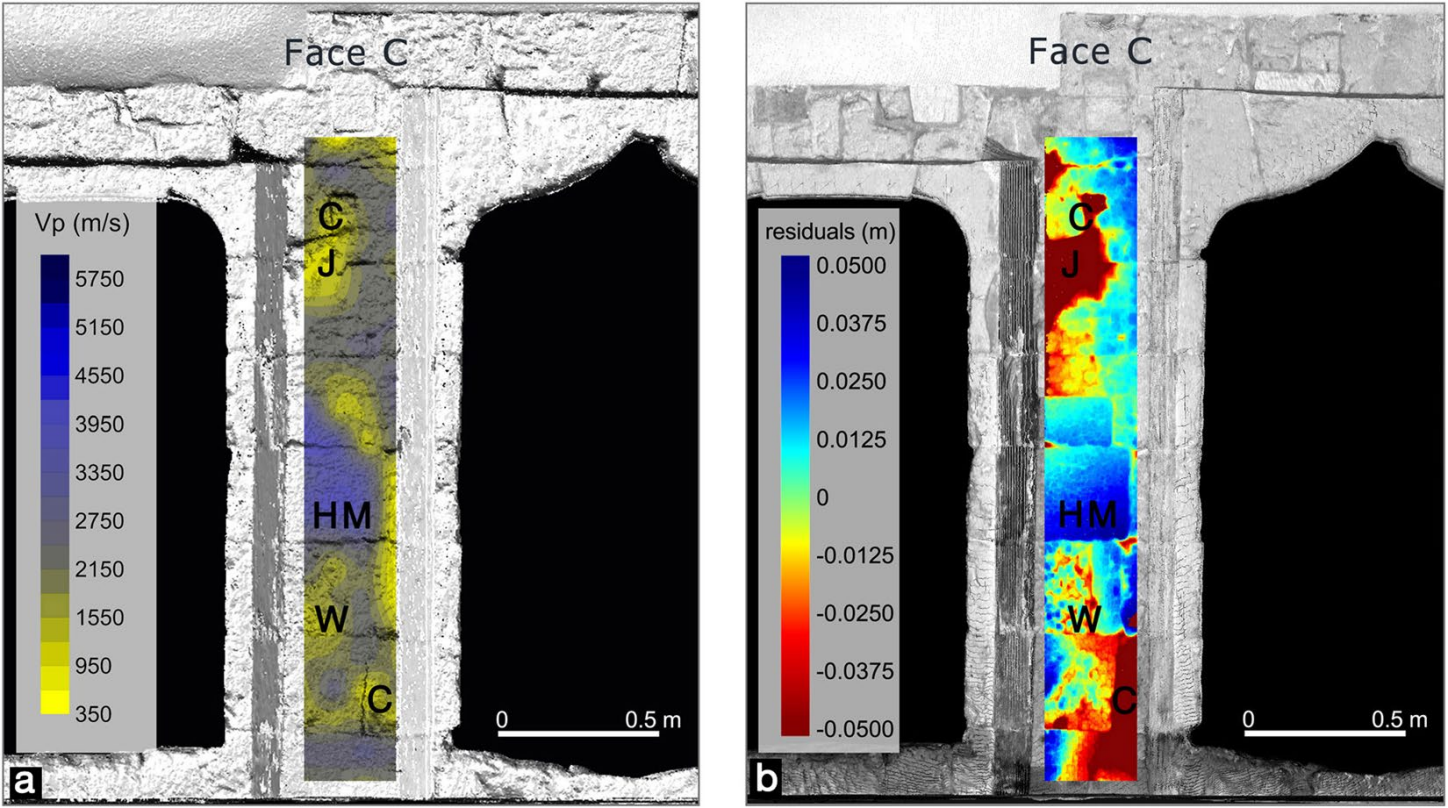
Comparison between the ultrasonic map and the TLS results on face C of the pillar. The two techniques of the NDT survey show matching results highlihted by uppercase letters. (C = crack; J = Junction; W = Weathering; HM = Health Material).

Moreover, we computed a high resolution 3D model of the ancient portal of the *Palazzo di Città* after selection of the point cloud that illuminates this particular architectural element, and in the best operating conditions for TLS i.e. short distances (0–3 m) and small inclination angles of the laser beam (<30°). After fine registration and aggregation with millimeter accuracy to compute the surface anomaly, we fitted in the least square sense multiple planes geometric primitives to the surface of the portal, and computed the residuals. Faces A and C of the pillar, represented respectively in Figs 5a,b and 6a,b are taken into account for comparison with the longitudinal velocity maps detected with the ultrasonic methods by the indirect/surface acquisition modality ISRM 1978 (International Society for Rock Mechanics)^21^. For the sake of convenience we projected the geometric anomalies on the corresponding areas of the surface of the 3D model of the portal texturized with the reflectivity. The resulting model is shown in Figs 5a,b and 6a,b and represents the faces A and C of the studied architectural pillar. As can be noticed in the above figures the geometrical surface anomaly varies in an interval of +/−5 cm, the positive anomaly occurs in correspondence of the unaltered zones of the surface of the material, and is represented in blue (letters HM - Health Material), conversely the negative ones corresponding to the lowering parts of the surface of the pillar are represented in red (letters J, C, W in Figs 5b and 6b). Looking at the Figs 5b and 6b it is evident that red colored surface anomalies are present in correspondence of the joints between blocks where voids are visible, and in correspondence of fissures or collapses of the material used to assemble the pillar. These anomalies are highly correlated with the trend of shallow anomalies detected with the ultrasonic tests.

As a matter of fact despite its high productivity the TLS methodology can only monitor the surface anomaly of an illuminated body, and for this reason a comparison with methodologies, such as ultrasonic ones, that have a capacity of investigation in its inner parts is needed for a more complete diagnostics of the conservation status of the investigated architectural element.

The ultrasonic longitudinal velocity maps obtained with the indirect transmission mode (transmitter and receiver on the same face of the investigated architectural element) give a clear picture of the elastic conditions of the shallow building materials. Velocity variations detected in the longitudinal velocity maps on the faces of the pillar (Figs 5a and 6a) are to be interpreted as zones of variation in the petrographical characteristics (especially texture) and/or zones of different elastic-mechanical conditions. The presence of voids, fissures, discontinuities and alterations causes a slower propagation of the elastic waves as they attenuate the ultrasonic signal by absorbing its energy.

In Fig. 5a the ultrasonic longitudinal velocity map on face A of the pillar obtained by indirect modality and superimposed to the TLS surface geometric anomalies shows different low velocity zones (yellow areas) along the vertical development of the pillar. In the upper part (zone J, Fig. 5a), the low velocity zone can be associated to a discontinuity (junction) filled by thin mortar between the carbonate stone blocks. The other low velocity zones, marked in yellow, at different levels of the longitudinal development of the pillar, can be related to the presence of defects like fissures and cracks (zones C, Fig. 5a) on the ashlars, that are caused by natural or artificial causes. The other low velocity zones can be related to the physical-chemical weathering (zones W Fig. 5a). In Fig. 6a the ultrasonic velocity map on face C of the pillar also shows many low velocity zones, which can be associated to cracks (zones C Fig. 6a) or physical-chemical weathering (zones W, Fig. 6a). Moreover, the geometrical anomalies detected by TLS technology (Figs 5b and 6b) show significant analogies with the ultrasonic longitudinal velocity maps discussed above. In general, the low velocity zones in faces A and C of the pillar (Figs 5a and 6a) are associated to the TLS negative geometrical anomalies where loss of material or morphological irregularities due to cracks or chemical-physical weathering occur. The high velocity zones (blue color) correlated to health material (zones HM Figs 5a and 6a) correspond to the TLS positive anomalies (Figs 5b and 6b). These are in correspondence of the unaltered parts of the surface of the material where no defects or weathering phenomena occur.

**Figure 7:**
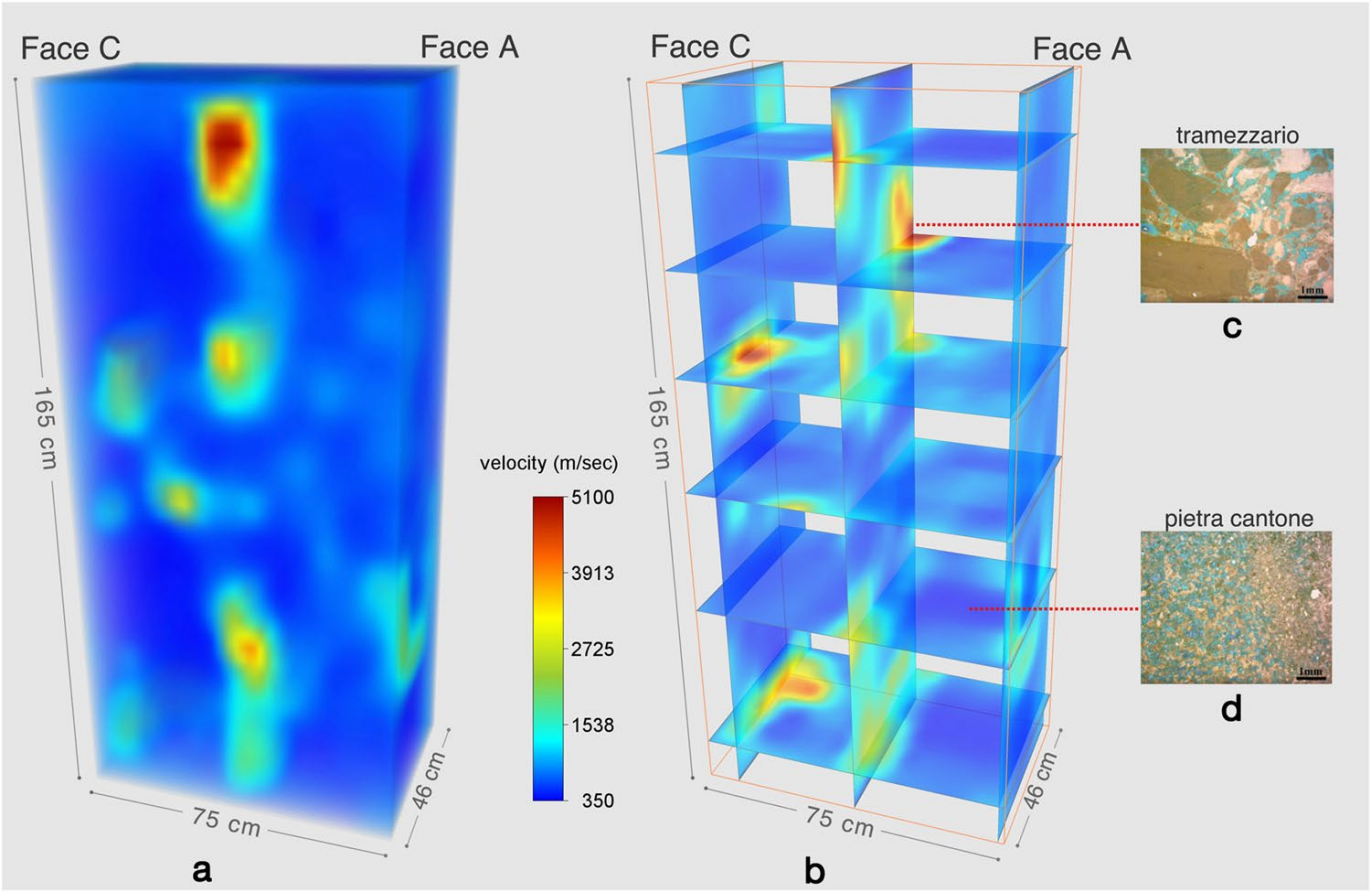
(**a**) 3D tomography of the pillar; (**b**) 3D view of tomographic slices; (**c**) thin section of the *Tramezzario* showing its textural characteristics, dominant texture: grainstone; (**d**) thin section of the *Pietra Cantone*, dominant texture: mudstone. The thin sections were impregnated with blue epoxy to better visualize the pore space. 3D data represented with Voxler-3D geologic and scientific modeling software (Golden Software), http://www.goldensoftware.com/.

From the ultrasonic tomography, a 3D reconstruction of the longitudinal velocity distribution inside the pillar was produced (Fig. 7a). The longitudinal velocity (Vp) inside the building materials presents high variability (from 350 m/s to 5100 m/s), which denotes that the elastic characteristics of the carbonate materials are greatly heterogeneous. The longitudinal velocity distribution inside the pillar, its comparison with the velocity laboratory measurements^1,11^ and the in-depth knowledge of the rock petrography, allow to reasonably conclude that the pillar is made up mainly of *Pietra Cantone* carbonate rock and subordinately of *Tramezzario* carbonate rock. In these rocks, grains, matrix and cement include soluble components, which favour the changes that affect the ultrasonic signal propagation. In the *Pietra Cantone*, the pore types that were classified based on SEM analysis are mainly “interparticle” of primary origin^19^. The effect of this porosity on the longitudinal velocity propagation is that the moisture water-filled pore space can cause a slowdown of the ultrasonic wave propagation. In fact, in a mud supported carbonate rock, such as *Pietra Cantone*, the presence of water or moisture causes a decrease in the cohesion forces among the carbonate mud particles, which leads to a decrease in compactness. The water within the pores interacts with the material and progressively dissolves the calcite, making the rock extremely vulnerable and prone to alteration and dissolution phenomena. The consequence of this is a worsening of the elastic condition of the rock. These characteristics can justify the low velocity that characterizes the carbonate building materials inside the pillar. A few tomographic slices (Fig. 7b) through the corresponding longitudinal sections of the investigated pillar were selected on account of the presence of building material heterogeneity. The three dimensional view of the slices (Fig. 7b) favours the diagnostic analysis by showing the depth, size and geometry of areas at different elastic conditions. Considering the laboratory test on the physical properties of the same materials described in Cuccuru *et al*.^1^ and Fais *et al*.^11^, and the petrographical analyses carried out in this study, the few higher velocity areas (marked in brown-red) encased in the *Pietra Cantone* low velocity carbonate material, can be related to the *Tramezzario* carbonate rock. This material is grain supported and the presence of cement (sparry calcite) provides an excellent bonding between the bioclasts, which inhibits the granular disaggregation and makes this rock less porous (Fig. 7c) and more compact than *Pietra Cantone* (Fig. 7d), thus enhancing its elastic properties.

From the above discussion it can be deduced that our procedure can give a detailed diagnostic analysis of the building materials of architectural structures that can lead to a successful precise recognition of the characteristics of both shallow and inner parts of the material. This task is highly performing and of useful application in areas where both ancient and modern buildings are affected by natural disasters, such as earthquakes, landslides, noticeable amount of land subsidence.

## Conclusions

In this work we demonstrate that the integration of complementary information can improve the diagnostic process on the conservation status of the stone building materials.

As a matter of fact the stone building materials are very complex to analyze due to the heterogeneity of their intrinsic characteristics. For this reason in many situations this must be done with a high level of complexity in the diagnostic process. In fact, in the Cultural Heritage field the greater part of recent scientific work is targeted at the integration of heterogeneous datasets and complementary methodologies that can reduce diagnostic uncertainties as is the case in medicine. In synthesis, especially in complex situations, such as the analysis of the conservation status of heterogeneous stone materials, the use of a single methodology is generally unsatisfactory to perform a very accurate diagnosis.

An accurate study of the materials making up the analyzed artifacts with the aid of electronic and optical microscopy must be followed by 3D modelling with TLS and *in situ* and laboratory ultrasonic tests aimed to carefully analyze also the inner parts of the building materials.

In particular, in our study the integration of different methodologies allowed us to analyze in detail the conservation status of an architectural structure of historical importance.

The ultrasonic methods applied with different acquisition and processing techniques have been very effective in detecting the elastic conditions of the shallow and deep materials of the investigated structure. The response of this method was supported by the petrophysical analysis of the investigated stone materials such as petrographical composition, textural characteristics like type of porosity, grain disposition, and presence of cement or matrix between grains. The information deduced by the application of the TLS technique was an effective complement to ultrasonic data for a more complete understanding of the conditions of the shallowest part of the building materials of the investigated structure.

## Materials and Methods

### Materials

The stones used as building materials of the monumental structures of Cagliari come from a carbonate succession that crops out in the town hills and is known as “Calcari di Cagliari auct”^22,23^. This sedimentary complex is attributed to the Tortonian- Messinian^24^ and is formed of three different carbonate rock types from top to bottom called: *Pietra Forte*, *Tramezzario* and *Pietra Cantone*.

*Pietra Forte* is a massive, well lithified biohermal and biostrome limestone, formed in a high energy environment with a paleobathymetry of less than 30 m^25^. It is a very compact stone though it can occasionally be intensely fractured and affected by numerous karstic cavities.

*Tramezzario* is a well lithified bioclastic limestone related to a paleobathymetry of around 40 m^25^, and a high energy zone^26^. Locally, the degradation processes are facilitated by the presence of carbonate mud. In high humidity conditions a decrease of cohesive forces among micrite particles causes pulverization phenomena, making Tramezzario assume the characteristics of a loose rock.

*Pietra Cantone* is a poor lithified, bioclastic, frequently bioturbated limestone, that formed in a paleoenvironment about 60–80 m deep^25^. It is a very soft stone, characterized by high porosity and by a high percentage of matrix, making this rock very hygroscopic.

Some transition facies are present among these lithothypes. They present mixed textural features due to variations in the depth and energy level of the sedimentary environment.

### Methods

#### Petrographic analyses

In order to determine the petrographic characteristics of the “Calcari di Cagliari” Formation, several samples of these limestones have been analyzed from a textural and mineralogical point of view by means of thin sections observed on an optical petrographic microscope (OPM) and scanning electron microscope (SEM). For a better identification of the pore network at the OPM, thin sections were stained with an epo blue resin^18^. The mineralogical-petrographic study of the above carbonate rocks was carried out to correlate their textural features with the propagation of the longitudinal ultrasonic waves and with the reflectivity and geometrical surface anomalies detected with TLS methodology. In addition to textural characteristics, it is important to analyze the nature and distribution of porosity. An understanding of the textural and physical characteristics of the study carbonate materials is necessary for the interpretation of the ultrasonic data collected in the investigated architectural pillar.

#### 3D Terrestrial Laser Scanner

Methodology: TLS is a proximal sensing technology that allows to 3D model objects of complex shapes like ancient building and artifacts, but also monitor landslides, caves, volcanoes and other environmental phenomena.

In modern laser scanners a semi-conductor laser beam is projected on a regular grid by means of systems of mirrors driven by automated engines.

To 3D model the investigated artifacts we operate long-range distance-meter laser scanners whose phase comparators can routinely measure a distance between an arbitrary point inside the sensor and the target point illuminated by the scanner, the distance being proportional to the phase difference between the emitted pulse and the reflected one^8,10,11^.

The distance and direction of the amount of laser pulse reflected and then detected by the sensor are converted into Cartesian coordinates of the pixels (picture elements) composing the area of the investigated objects. The result of a 3D TLS survey are several point clouds, one or more of them for every station point. For each point of these clouds, the coordinates are made available in an arbitrary intrinsic reference frame inside the sensor and when available the texturing is in general expressed as three integer numbers, i.e. the radiometric values, in a Red-Green-Blue (RGB) color scale. Moreover, a reflectivity parameter is always provided and defined in first approximation as the normalized ratio between the amount of energy reflected by the targeted surface points and the energy emitted by the laser sensor^8,9,11^.

Modern instruments used for architectonic studies can acquire clouds of millions of points in a few seconds and are characterized by both a horizontal and a vertical Field of View (FOV), in fact modern LS can observe angles as wide as 340° × 360°.

The 3D model of complex shape objects like buildings or artifacts (walls, columns, pillars) are computed by aggregating several point clouds collected by the TLS installed in different station points all around the building prospect and internal parts in order to illuminate all the elements composing a complex shape body to form a unified unstructured registered point cloud (3D model).

Instrumental data: As part of the 3D modeling survey we tested and operated a Leica model HDS-6200 phase shift long-range laser scanner. The instrument has vertical Field of View (FOV) on a maximum distance range of 79 m, a nominal precision of 1 mm for distances <=25 m, and a divergence angle of about 0.22 mrad (i.e. 0.22 mm/m).

By computing the 3D model of the *Pietra Cantone* ancient portal of the *Palazzo di Città* Museum, several tenths of point clouds were acquired using the highest precision modality. In a survey lasting a few days, more than 100 million points were gathered, and for each measured point Cartesian coordinates X, Y and Z, and the reflectivity parameter normalized in the range 0–1 were made available.

A number of rooms and internal parts of the ancient palace hosting the museum were surveyed and the corresponding point clouds were pre-processed, processed and unified, together with the external walls and the facade to obtain the high resolution 3D model^11^.

TLS data processing: The 3D TLS data processing procedure can be summarized in the following steps: data preprocessing and registration, 3D surfaces precise modeling, computation of the geometrical anomalies (Fig. 4a,b).

During the data pre-processing step, the data were automatically filtered and manually edited in order to remove the main error sources and data out of tolerance. In a second step, the registration process was applied to define a unique intrinsic reference frame for all point clouds and to unify them^8,10,11^.

The first step of the registration procedure is an operation of cloud to cloud registration that is performed to draft align all the clouds. In a second step a bundle adjustment automatic procedure is carried out based on the Iterative Closest Point (ICP) algorithm^27,28^. At this stage particular care is necessary to minimize the errors of the alignment process at the centimeter level or better to improve the accuracy of the resulting unified registered point cloud.

In the following we describe a diagnostic procedure based on the critical analysis of the surface geometrical anomalies of the architectural parts of ancient buildings such as facades, walls, columns etc. The anomalies maps are obtained simply for a comparison of the investigated building parts with graphical primitives like planes, cylinders and spheres suitably fitted to the building elements to be analyzed, and to compute the residuals projected on a regular grid^10,11^ (Figs 5 and 6). In particular, we computed the geometrical anomalies of the surface of the ancient *Pietra Cantone* portal starting from the high resolution 3D model and by comparing it with a multi-plane geometry.

#### Ultrasonic Test

A portable Ultrasonic Non-Destructive Digital Indicating Tester (PUNDIT LAB PLUS) device by PROCEQ (Switzerland) with 24kHz-54kHz piezoelectric transducers was used to acquire ultrasonic data in the investigated pillar using different acquisition modalities, following ISRM (1978)^21^ and Normal 22/86^29^: indirect-surface transmission (transmitter and receiver on the same face of the pillar) to analyze the elastic conditions of the shallow parts of the building materials and direct transmission (transmitter and receiver on the opposite faces of the pillar) in order to detect any mechanical discontinuities or altered and damaged zones inside the investigated structure. The PUNDIT PLUS device was coupled to a portable oscilloscope (Fluke 96B) to acquire and digitalize the ultrasonic waveforms, and allow them to be analyzed and processed. The PUNDIT pulser was used to excite the P wave emitting transducers and to trigger the time base of the Fluke 96B digital oscilloscope. On traversing the investigated material, the longitudinal ultrasonic pulse was detected by the receiving transducer. Then it was amplified by a broad-band differential amplifier and displayed and stored in the memory of the Fluke digital oscilloscope. The transit time of the displayed ultrasonic signal was used to calculate the velocity of the longitudinal wave propagation. For each *in situ* measurement point, both in indirect and direct transmission, three consecutive observations were performed to check the accuracy in detecting the arrival time and then in calculating velocity. For each measurement, the repetition of the arrival time was satisfactory (differences between 0.125 μsec and 0.150 μsec).

Silicone snug sheets were used as a coupling agent because they were judged a better coupling agent than vaseline^30^ and because they have the great advantage of not leaving a trace on the monument. The 24kHz-54kHz frequencies were respectively used for the tests in direct and indirect transmission modes. They were selected based on different tests and in each case (direct and indirect transmissions) considered an acceptable compromise between the resolving power and a tolerable attenuation. As is known there are many factors that affect the reduction in the wave propagation energy and the velocity changes. However those that cause a greater reduction in the longitudinal velocity are linked to the pore geometry, boundaries between grains and presence of inhomogeneities such as cavities or cracks^31^. The signal scattering and energy absorption at the grain boundaries, cavities and cracks in the carbonate rocks can take place in different ways to produce an attenuation of the energy during signal propagation. But, in this regard, as is known^32^ scattering depends mainly on the relation between the wavelength and the grain size. If the wavelength is much larger than the grain size as in the case of the ultrasonic frequencies used in the tests on stone materials (54kHz-24kHz) the scattering is generally negligible.

The first modality (indirect transmission) was used to acquire the signals that travel from transmitter to receiver in the shallow parts of the study pillar, and to analyse their transit time that was determined by locating the first break in the ultrasonic waveforms. The transit time of the longitudinal ultrasonic signal deduced from the first break of the first arrival was measured with a good resolution, 0.1 microseconds. The transit time is crucial because it determines the propagation velocity value. A small time shift can cause a relevant velocity shift considering that the time scale is expressed in microseconds. Therefore analyzing the ultrasonic waveforms, only first arrivals with shapes that looked undistorted were considered. The ultrasonic data acquisition by indirect transmission was carried out along five equidistant parallel profiles in the vertical direction for each face of the investigated pillar using an offset (transmitter-receiver distance) of 0.1 meters. Special care was taken to make measurements at the same level in all profiles. Starting from the measured travel time of the ultrasonic signal, the apparent longitudinal propagation velocity was calculated by dividing the distance between transmitter and receiver by the travel time at each observation point along the profiles. The *in situ* Vp measurements were compared with the laboratory determinations on intact rock samples from the main outcrops of the investigated carbonate rocks and from ancient quarry sites in the town of Cagliari, presented in previous works^1,11^. The values of the longitudinal ultrasonic velocity deduced from the *in situ* measurements were contoured to represent the longitudinal velocity distribution on each face of the pillar with the aim of detecting damaged and degradation zones in the shallow parts of the building materials by analyzing the velocity variations and comparing them with the TLS parameters as surface geometry anomalies and reflectivity. Ultrasonic signal characteristics and their propagation velocity change as the wave propagates through the carbonate materials with varying petrographical and elastic properties also as a result of degradation. Figures 5 and 6 show the longitudinal ultrasonic velocity maps of the different faces of the pillar superimposed to the TLS unified point cloud texturized with the reflectivity parameter.

In a further step of the diagnostic process, the second modality (direct transmission) was used to acquire the ultrasonic data in cross direction and following the well known tomographic acquisition scheme in order to check the conservation status of the materials inside the pillar. As is known non-destructive tests by means of ultrasonic tomography allow to locate defects and altered or damaged zones within the materials, while also determining their geometry^14^.

Transmitters and receivers at 24 kHz ultrasonic frequency were positioned to obtain a good coverage of the ray paths associated with longitudinal ultrasonic waves propagating through the materials. The 3D ultrasonic tomography was obtained considering exclusively the first arrivals of the longitudinal ultrasonic signals while taking into account that the first arrival is easily detectable in the wave train. Ultrasonic travel time tomography allowed to reconstruct the distribution of the ultrasonic longitudinal velocity inside the pillar. The ultrasonic tomographic reconstruction is a typical example of an inverse problem, in fact the method is aimed to produce an estimate of the spatial distribution of the analyzed acoustic parameter by observing its effect. The region to investigate has to be divided into a grid of cells (pixels or voxels), and a value for the analyzed p(x, y) acoustic parameter is determined for each element (pixel) in the grid. The value in each element corresponds to the value of p(x, y) in the neighborhood of that point allowing to describe the parameter distribution function. In our study, the cell size was chosen considering the needed resolution, number of rays and wavelength of the ultrasonic signals. We acquired a total of 576 rays, we therefore analyzed 576 travel times to reconstruct the velocity tomogram of the pillar.

In order to obtain a realistic starting velocity model as an input for the simultaneous iterative reconstruction technique (SIRT)^33,34^ to invert travel-time data and produce a 3D representation of the ultrasonic longitudinal wave velocity distribution inside the pillar, a methodology based on the cross-correlation function proposed by Fais and Casula^14^ was used. Therefore, the cross-correlation function was computed and used as a constraint to the SIRT tomography to include prior knowledge of the investigated sections. Figure 7a,b respectively represent the 3D tomographic image of the investigated pillar and a 3D view of the tomographic slices inside the pillar.

## Electronic supplementary material

Tomography of the Pillar

Tomography Slices

TLS 3D Model of the Pillar
